# Skull Metastasis With Orbital Invasion as a Primary Manifestation of Hepatocellular Carcinoma

**DOI:** 10.7759/cureus.55091

**Published:** 2024-02-27

**Authors:** Samer Jumean, Shatha Elemian, Hamid S Shaaban, Gunwant Guron

**Affiliations:** 1 Internal Medicine, Saint Michael's Medical Center, Newark, USA; 2 Internal Medicine, New York Medical College, Valhalla, USA; 3 Hematology and Oncology, Saint Michael's Medical Center, Newark, USA

**Keywords:** bone metastasis, skull metastasis, gastrointestinal oncology, orbital invasion, temporal skull lesion, hepatocellular carcinoma (hcc)

## Abstract

Hepatocellular carcinoma (HCC) is a common worldwide cancer with a poor prognosis despite treatment advancements. Patients typically exhibit signs and symptoms pertaining to the liver. Extrahepatic metastasis of HCC is documented to be as low as 5% of cases. Bone metastasis ranks third following lungs and regional lymph nodes. The typical locations for bone metastasis include the vertebral column, pelvis, femora, and ribs, with skull metastasis, being reported in less than 1.6% of cases. Herein, we describe a case of HCC presenting with skull metastases and orbital invasion as the initial manifestation.

## Introduction

Hepatocellular carcinoma (HCC) is the most prevalent primary liver malignancy, and risk factors include chronic hepatitis B and hepatitis C infection, nonalcoholic fatty liver disease, alcohol abuse, and exposure to toxic materials, such as aflatoxin [1]. Despite the development of advanced screening modalities, the majority of the cases are diagnosed in advanced stages [2]. Clinical presentation of patients with HCC varies from generalized weakness and right upper quadrant pain to signs of obstructive jaundice or bone pain due to bone metastasis [3]. Metastatic HCC accounts for 15% of all cases [4], and the most sites of metastases are the lungs, bones, and abdominal lymph nodes [4,5]. Herein, we describe a case of HCC that presented with skull metastases with orbital invasion as the initial manifestation. 

This article was previously presented as a meeting abstract at the 2023 New Jersey Chapter American College of Physicians Scientific Meeting.

## Case presentation

**Figure 1:**
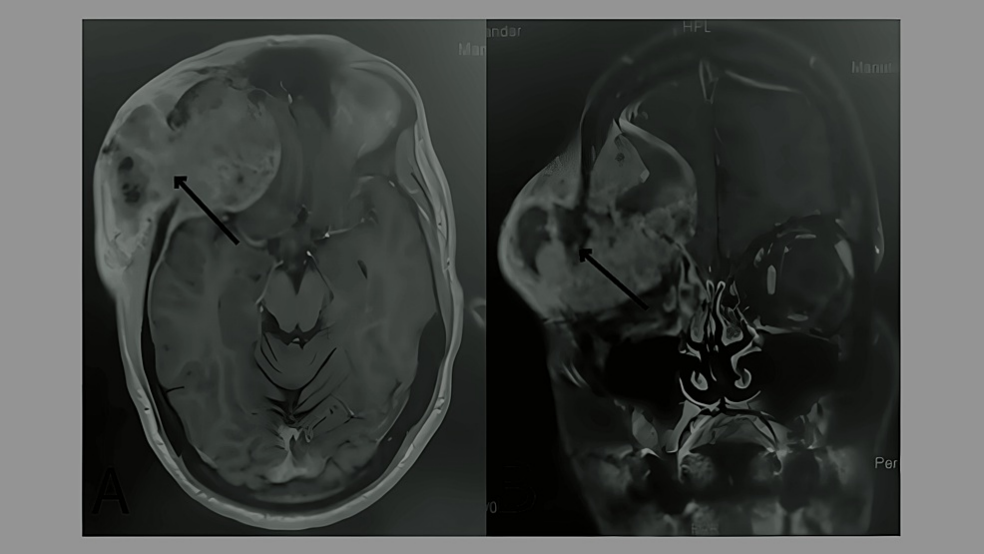
Axial and Coronal Sections of Brain MRI

**Figure 2:**
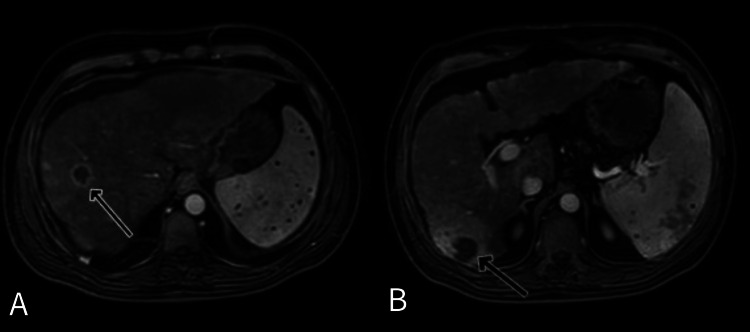
Liver MRI Showing Multiple Liver Masses

A 40-year-old male, known to have B-cell non-Hodgkin lymphoma in remission since the age of 15 and chronic untreated hepatitis C due to a lack of follow-up, presented complaining of a right temporal mass. Initially, the patient noticed a painless right temporal scalp swelling, which enlarged rapidly over several weeks and later became associated with right eye pain. He denied any history of head trauma. Within the following weeks, the patient experienced rapidly worsening symptoms, including right eye proptosis, eye pain, blurry vision, and headache. On physical evaluation, the patient exhibited gross deformity of the right upper face, significant right eye proptosis, fixed eye movements, and upper and lower eyelid erythema and edema, and no signs of liver cirrhosis on physical examination were noted. Brain MRI (refer to Figure 1) identified a 7.5 cm right frontotemporal destructive mass involving the sphenoid orbital region extending into intracranial and extracranial soft tissue. A biopsy demonstrated cells positive for hepatocyte-specific antigen (HSA), arginase, and glypicans 3, with a few cells positive for alpha-fetoprotein and CD10, which are specific for HCC. Laboratory findings are shown in Table 1. The serum alpha-fetoprotein level was notable at 1280. Retrograde diagnostic workups, including whole-body CT, were performed to identify a primary lesion. CT of the abdomen revealed significant liver cirrhosis with ill-defined enhancing lesions in hepatic segments 7 and 8 with no evidence of metastasis, ascites, or esophageal varices. Liver MRI (refer to Figure 2) showed liver cirrhosis with four right lobe lesions, with the largest in segment 7, measuring 2.5 cm. Peritoneal tapping did not reveal any malignancies. A diagnosis of hepatocellular carcinoma with metastasis to the skull and orbit was made. During the hospital course, the patient suffered from a sudden onset of massive upper gastrointestinal (GI) bleeding and passed away before receiving any treatments for HCC.

**Table 1:** Laboratory Findings on Admission WBC: White Blood Cells; ALT: Alanine aminotransferase; AST: Aspartate aminotrasnferase; ALP: Alkaline phosphatase; T.Bili: Total bilirubin

Laboratory measure	Value	Reference range
Hemoglobin	9 g/dL	13.5-16 g/dL
Platelet count	72,000 10^9^/L	150,000-450,000 10^9^/L
WBC	4,000 10^9^/L	5,000-10,000 10^9^/L
ALT	72 IU/L	8-33 IU/L
AST	50 IU/L	4-36 IU/L
ALP	369 IU/L	44-147 IU/L
T.Bili	1.2 mg/dL	0.1-1.2 mg/dL
Direct bili	0.60 mg/dL	Less than 0.3 mg/dL
Total protein	8.4 g/dL	6-8.3 g/dL
Albumin	4.3 g/dL	3.4-5.4 g/dL
Creatinine	0.8 mg/dL	0.7-1.3 mg/dL
Sodium	143 mEq/L	135-145 mEq/L
Alpha-fetoprotein	1280 ng/mL	0-40 ng/mL

## Discussion

HCC is identified as the fourth cause of cancer-related death globally [6], with high incidence rates in Asia and Sub-Saharan Africa [7], where the burden of chronic hepatitis B and C viral infections is heavy [8,9]. The implantation of advanced surveillance approaches and guidelines has significantly contributed to the early detection of HCC. Thus, early treatment with surgical resection and radiofrequency ablation leads to a decrease in mortality rates and an increase in the five-year survival rate [10,11]. This increase in survival rate and the utilization of advanced diagnostic techniques increased the observed incidence of extrahepatic metastatic lesions from primary HCC [4,12]. Extrahepatic metastasis is commonly present in patients with advanced disease (tumor size > 5 cm) [13]. Hematogenous spread due to angiogenesis and microinvasion of blood vessels is the most described way of metastasis; due to the presence of portal hypertension, the spreading of malignant cells through portal-vertebral veins plexuses could occur [14], which explains the high incidence of bone metastasis. Cranial metastasis could occur through an osseous pathway via Bateson’s venous plexus, which was proposed by Yen et al. [15]. Bone metastasis of HCC is the third most common extrahepatic site of metastasis following the lungs (34-70%) and regional lymph nodes (16-40%). Fukutomi et al. [14] reported that the bone metastasis from HCC incidence had increased to 13%, and the usual sites of bone metastasis are the vertebral column, pelvis, femora, and ribs, with less than 1.6% of reported cases reported within the skull [16]. It has been shown that skeletal metastasis, including the skull, is increasing, relating to the progress in diagnosis and treatment [14]. The diagnosis of HCC is often made with advanced disease when patients have become symptomatic and have some degree of liver impairment. At this late stage, there is virtually no effective treatment that would improve survival [4]. In a review by Hsieh et al., the most common clinical manifestations of HCC skull metastasis were found to be a subcutaneous mass with occasional painful sensation (57%), neurological deficits and cranial nerve palsies (51%), headache (15%), and seizures [17]. In our case, the initial symptoms were eye pain and a headache attributed to the mass effect. Furthermore, HCC-affected patients have concomitant liver dysfunction, which can lead to decreased production of the coagulation factors and subsequent coagulopathy, which may result in bleeding from the skull metastasis, such as epidural hematoma. This bleeding would be difficult to control and may ultimately lead to death [18]. Among a narrative review of 24 cases with skull metastases as the first symptom of HCC, 17 of the 24 cases were misdiagnosed due to the lack of proper attention to the increasing incidence of skull metastasis of abdominal malignancies, including HCC [16,19]. Further supporting the clinical manifestations, radiographic imaging helps in the diagnosis of skull metastasis. Radiological findings on X-ray or CT scans are destructive or osteolytic lesions or could present as a hyper-vascular mass in angiography [20]. However, MRI will be better for the evaluation of skull-based metastasis as it is more sensitive in the detection of soft tissue lesions, as in our case, especially when the fat suppression technique and gadolinium contrast are used [21]. The definitive diagnosis, however, would be made by taking a biopsy from the skull lesion. The way we approach treatment of skull metastasis from HCC should be individualized according to the part of the skull that is involved, tumor size, and the patient’s general condition. However, since skull metastasis represents an advanced stage of HCC, the treatment is mainly palliative. The goals of treatment are to relieve the pain and reduce the risks of neurological sequelae, thus improving or maintaining the quality of life. Treatment modalities could include radiotherapy and surgery, as applicable [14,22]. In the case of highly vascular lesions, trans-arterial embolization before surgery can be attempted [20,23-25].

## Conclusions

In conclusion, this case demonstrates a rare presentation of a patient with a lump causing headaches and eye pain, caused by HCC-associated skull metastasis. The presentation of malignancies is often vague and unpredictable, and it may lack alarming symptoms, which leads to late presentations. We should consider ectopic HCC as a rare possibility in a heterogeneously enhanced solid tumor observed anywhere in the body, associated with high alpha-fetoprotein values.
